# Lengthening Reconstruction Surgery for Fibular Hemimelia: A Review

**DOI:** 10.3390/children8060467

**Published:** 2021-06-02

**Authors:** Corey B. Fuller, Claire E. Shannon, Dror Paley

**Affiliations:** 1 Department of Orthopaedic Surgery, Loma Linda University, Loma Linda, CA 92354, USA; CoreyFullerMD@gmail.com; 2 Paley Orthopedic & Spine Institute, St. Mary’s Hospital, West Palm Beach, FL 33407, USA; CShannon@Paleyinstitute.org

**Keywords:** fibular hemimelia, SHORDT, SUPERankle procedure, Paley classification, leg length discrepancy

## Abstract

Fibular hemimelia (FH) presents with foot and ankle deformity and leg length discrepancy. Many historic reconstructions have resulted in poor outcomes. This report reviews modern classification and reconstruction methods. The Paley SHORDT procedure (SHortening Osteotomy Realignment Distal Tibia) is designed to correct dynamic valgus deformity. The Paley SUPERankle procedure (Systematic Utilitarian Procedure for Extremity Reconstruction) is designed to correct fixed equino-valgus foot deformity. The leg length discrepancy in FH is successfully treated with serial lengthening and epiphysiodesis. Implantable intramedullary lengthening devices have led to all internal lengthenings. Recent advancements in techniques and implants in extramedullary implantable limb lengthening (EMILL) have allowed internal lengthenings in younger and smaller patients, who would traditionally require external fixation. These new internal techniques with lengthenings of up to 5 cm can be repeated more easily and frequently than external fixation, reducing the need to achieve larger single-stage lengthenings (e.g., 8 cm). Modern reconstruction methods with lengthening are able to achieve limb length equalization with a plantigrade-stable foot, resulting in excellent functional result comparable or better than a Syme’s amputation with prosthetic fitting.

## 1. Introduction

Fibular hemimelia (FH) is the most common lower-extremity congenital longitudinal deficiency, with occurrence between 1:135,000 and 1:50,000 births. It is associated with a constellation of deformities that fall into five general categories: tibial deformity, genu valgum, knee instability, leg length discrepancy (LLD) and foot and ankle deformities and deficiencies. Both deformities and leg length discrepancy in FH can present with a wide spectrum from mild to severe [1,2,3].

In addition to being hypoplastic, the tibia often presents with a diaphyseal valgus-procurvatum deformity with a skin dimple over the apex of angulation. Independent of the tibial diaphyseal valgus, the knee joint often presents with valgus orientation as well, which can originate in the proximal tibia, distal femur or both [4]. The cruciate ligaments can be hypoplastic or aplastic, creating anterior and posterior knee instability [5]. This is often asymptomatic in childhood but can become more problematic as the child grows.

The majority of FH cases present with unilateral involvement and are associated with a leg length discrepancy that originates from growth inhibition of the tibia and foot. Many children with FH have associated congenital femoral deficiency as well, in which femoral growth inhibition contributes to the overall leg length discrepancy. The LLD at skeletal maturity arising from the tibia in FH and can be minimal to severe (25 cm). Combined with femoral inhibition this can become greater than 30 cm. The LLD at skeletal maturity is predictable using standard prediction methods including the Paley multiplier method and it follows the Shapiro 1a curve [6,7,8,9,10].

The foot and ankle deformities in FH have traditionally been the most challenging and disabling problems. In addition to absent rays and bracket metatarsals and syndactyly, the foot in more involved cases often presents with rigid and severe equino-valgus deformity. This fixed equino-valgus originates from a dysplastic and valgus distal tibia, a subtalar coalition malunited in equino-valgus, or both. The combination of rigid equino-valgus with a significant LLD has historically resulted in poor outcomes from reconstruction, resulting in ablative surgery commonly being recommended.

## 2. Classification

**Figure 1:**
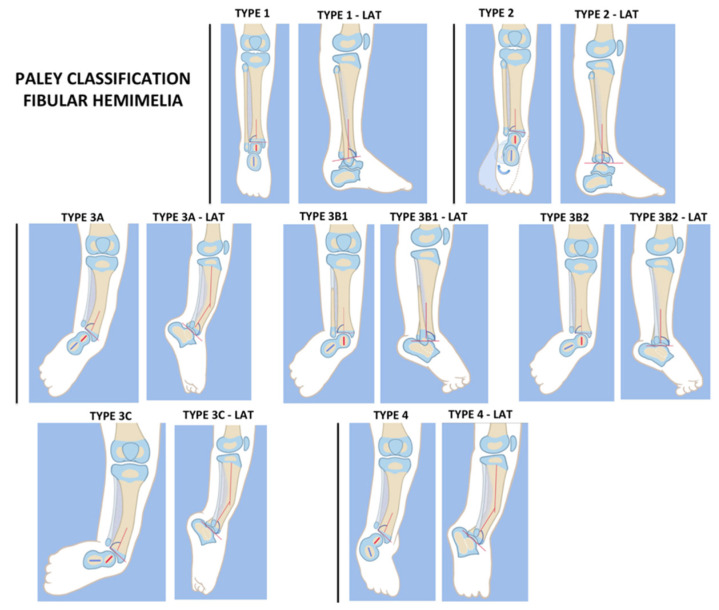
Paley classification for fibular hemimelia (FH), type 1: stable ankle, type 2: dynamic valgus ankle, type 3: fixed equino-valgus ankle, type 3A: ankle type, type 3B: subtalar type, type 3C: combined ankle/subtalar type, and type 4: fixed equino-varus ankle. LAT, lateral. Reproduced with permission by the Paley foundation.

Numerous classifications have been described, that are largely descriptive and focused on the fibular pathology, which does not require reconstruction. Many were created at a time when ablative surgery with prosthesis was the only reliable surgery option [11,12,13,14,15,16,17]. The Achterman and Kalamchi classification categorizes fibular hemimelia into three types: type Ia—fibular hypoplasia; type Ib—partial absence; type II—complete absence. Since degree of foot deformity or leg length discrepancy is not related to degree of fibular absence, and since treatment cannot be guided by degree of fibular absence, this descriptive classification of fibular deficiency is not useful for guiding limb reconstruction or even for comparison of cases [17,18]. The Paley classification (Figure 1) was designed by the senior author (D.P.) and was developed with the tibial pathology and foot and ankle deformity in mind, as these are the focus of reconstruction [15,16].

## 3. Radiographic Examination

Radiographic workup should include full-length anteroposterior (AP) standing radiograph with the patellas pointing forward. A lift of a measured amount should be placed under the affected limb to closely equalize the leg lengths to improve overall measurement accuracy. In young children who are not yet able to stand, a supine radiograph can be utilized. A long leg lateral radiograph with the knee in maximum extension is required to both increase the accuracy in length measurements and to evaluate for flexion contractures or subluxation of the knee.

## 4. Magnetic Resonance Imaging and Computed Tomography

**Figure 2:**
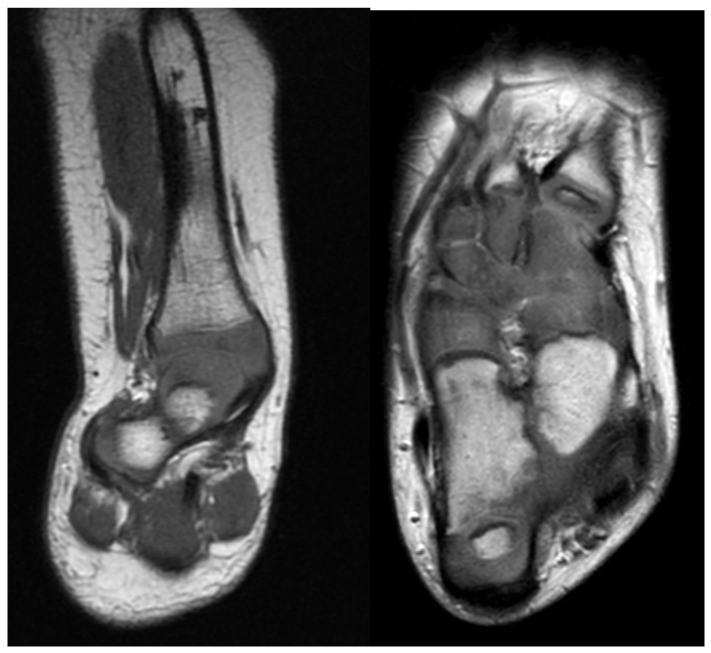
Left is coronal cut of magnetic resonance imaging (MRI) through the ankle and subtalar regions. This shows a subtalar coalition with the Calcaneus to the side of the talus. The ankle has a ball and socket shape. Right is axial cut of MRI through the foot showing the malunited talo-calcaneal coalition with the calcaneus to the side of the talus. The talonavicular and calcaneocuboid coalitions are also seen.

**Figure 3:**
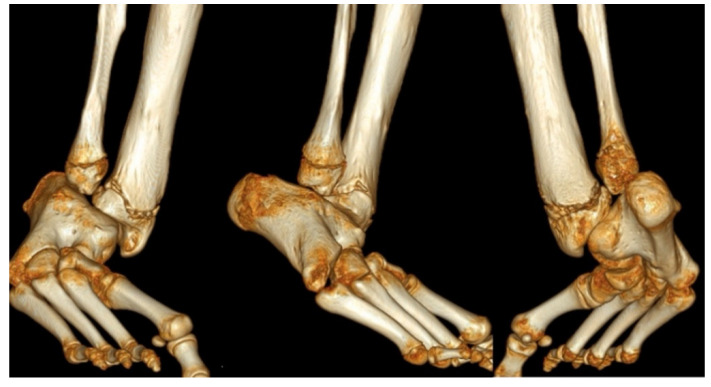
A three-dimensional CT scan of distal tibia and foot (left Anteroposterior (AP) view, middle lateral view and right posteroanterior (PA) views) in a 13-year-old boy with untreated type 3c fibular hemimelia with fixed equino-valgus of the hindfoot. Despite the presence of a fibula, there is valgus-procurvatum malorientation of the ankle plafond, and a malunited subtalar coalition. The calcaneus is articulating with the fibula. There are also calcaneo-cuboid and talo-navicular coalitions with midfoot adductus.

Magnetic resonance imaging (MRI) is not necessary in FH to differentiate between Paley types 1, 2, 3 or 4. This can typically be easily classified with clinical and radiographic examination only. An MRI becomes helpful in subclassifying Paley type 3 FH between a, b, or c and Paley type 4 by visualizing the mal-orientation of the tibial plafond present in types 3a and 3c and the malunited subtalar coalition in types 3b, 3c and 4 (Figure 2). It can also be helpful to identify aberrant vasculature [19,20] and the proximity of the posterior tibial vessels to the fibular anlage. Although it can also identify intra-articular pathology at the knee such as deficiency of the cruciate ligaments, this is best determined by physical examination. Computed tomography can be useful in older children whose bones are more ossified (Figure 3).

## 5. Foot and Ankle Reconstruction

The Paley classification is independent of the number of rays and LLD. Each Paley type is accompanied by a reconstructive treatment for the tibia, foot and ankle deformities. Most cases of Paley type 1 FH do not need any foot or ankle surgery before lengthening as the ankle is stable. In contrast, most cases of Paley type 2–4 FH will need foot and ankle reconstructive surgery to stabilize and/or create a plantigrade foot. Foot and ankle reconstruction usually needs to be performed prior to or can be combined with the first lengthening.

**Figure 4:**
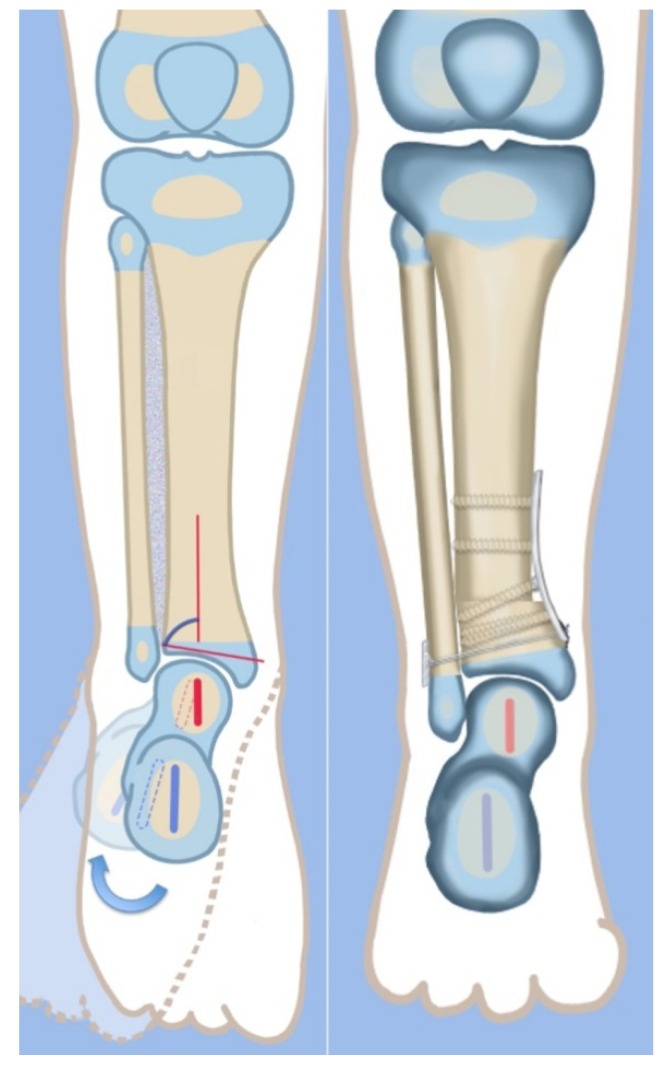
Illustrations before (**left**) and after (**right**) the SHORDT procedure (SHortening Osteotomy Realignment Distal Tibia) for dynamic valgus deformity of Paley type 2 FH. The main elements are the shortening and varusization of the tibial plafond relative to the fibula which does not change length. This eliminates the valgus instability of the ankle joint.

The SHORDT procedure (SHortening Osteotomy Realignment Distal Tibia) was designed by the senior author (D.P.) in 2014 to treat valgus instability of the ankle in patients who have a hypoplastic fibula with a distal fibula physis present [18] (Figure 4). This is commonly found in Paley type 2 FH. The SHORDT procedure involves a shortening and realignment tibial supramalleolar osteotomy to correct ankle valgus and procurvatum malorientation and lengthen the fibula relative to the tibia. This often results in a trapezoidal segment of bone being removed from the tibia and requires taking down the ankle syndesmosis before shortening the distal tibia. By shortening the tibia relative to the fibula, the fibula is effectively lengthened, restoring the buttressing effect of the lateral malleolus against dynamic ankle valgus. This addresses the foot and ankle deformity and instability in Paley type 2 FH and prepares the patient for concomitate or future leg lengthening. The SHORDT procedure does produce an acquired leg length discrepancy by the amount shortened that must be accounted for in future limb equalization.

**Figure 5:**
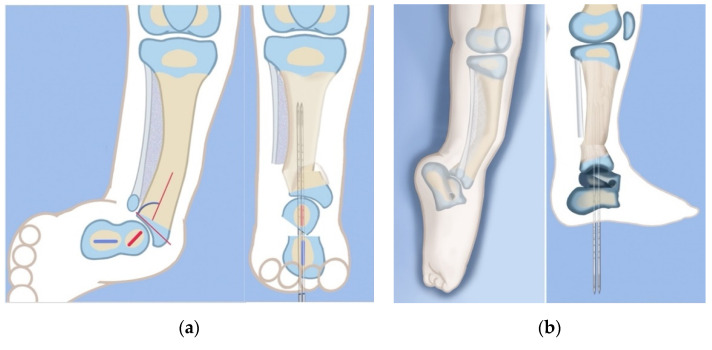
Illustrations of before (**left**) and after (**right**) the SUPERankle procedure (Systematic Utilitarian Procedure for Extremity Reconstruction) procedure for fixed equinovalgus deformity of Paley type 3C FH from AP (**a**) and lateral (**b**) views. The main elements of this procedure are the subtalar angulation translation osteotomy and the supramalleolar varus-extension with shortening osteotomy.

**Figure 6:**
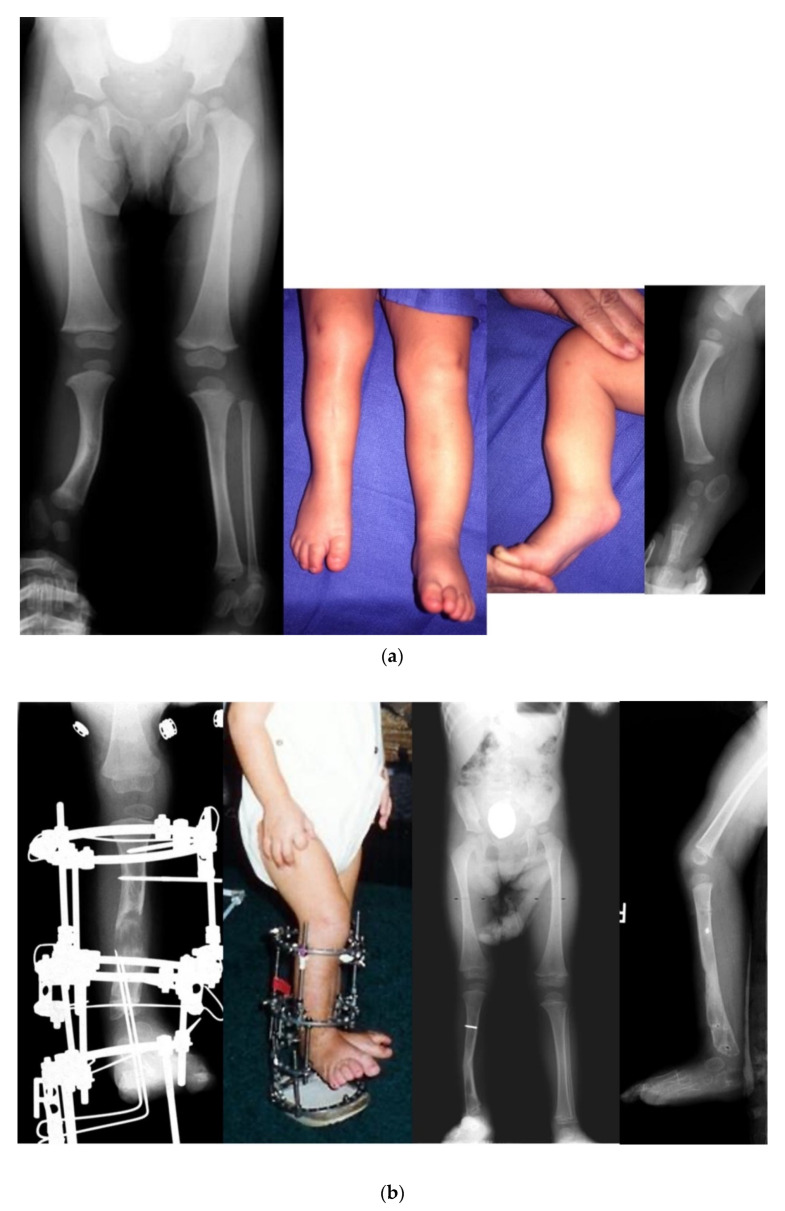

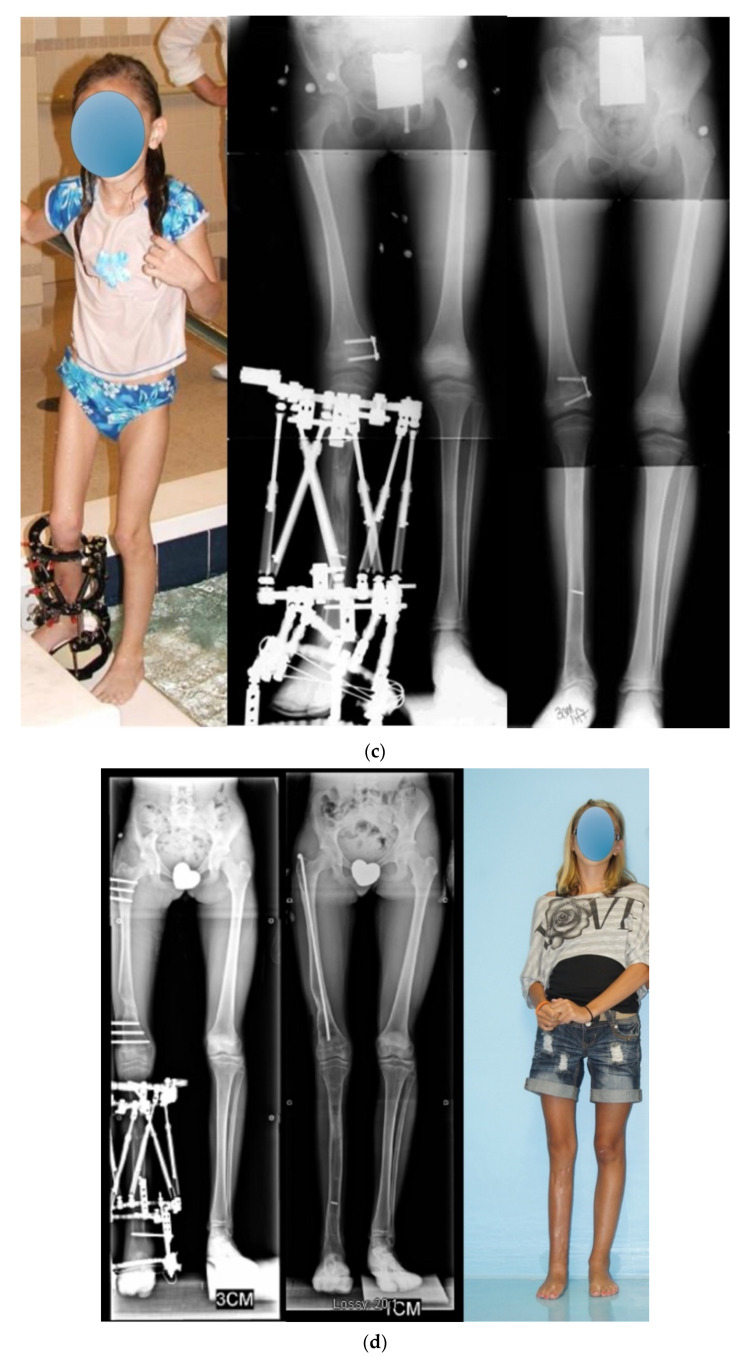
(**a**) Preoperative radiographs and photographs of an 18-month-old girl born with Paley type 3a FH. She has fixed equinovalgus of the foot and a procurvatum-valgus diaphyseal tibial deformity. (**b**) Photograph (**left**) and radiograph (**middle left**) after the SUPERankle procedure combined with application of a circular external fixator for 5 cm of lengthening. Radiographs of the lower limbs after removal of the external fixator (**right middle** and **right**). (**c**) Photograph (**left**) and radiograph (**middle**) showing second lengthening of tibia at age 8, using computer dependent external fixator. She is shown doing pool therapy. Radiograph after removal of external fixator with correction of distal femoral valgus malalignment by hemiepiphysiodesis (**right**). (**d**) Radiograph showing the third and final lengthening of the tibia with lengthening of the femur both with external fixation at age 13. Radiograph after removal of the external fixators showing excellent alignment and equalization of limb lengths (**middle**). Final photograph at age 16 years after skeletal maturity with equal leg lengths and excellent function.

**Figure 7:**
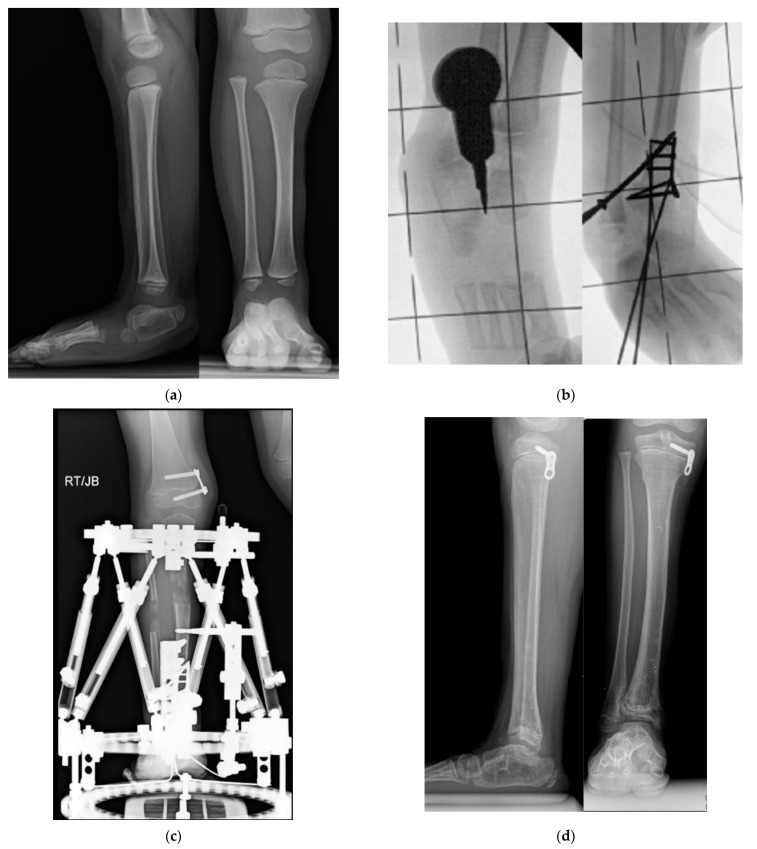

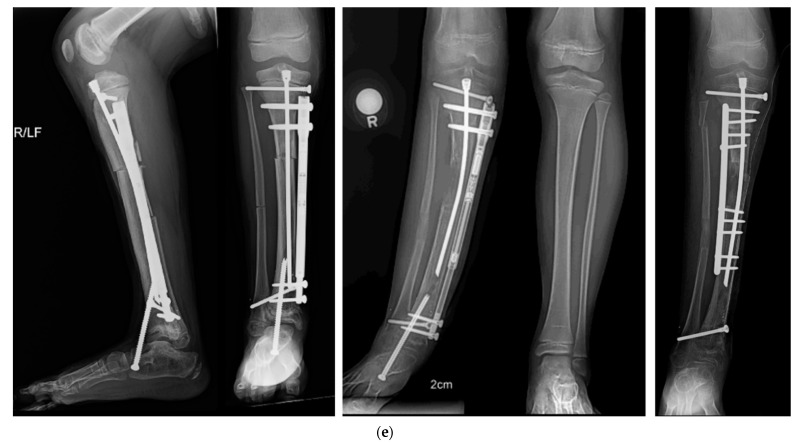
(**a**) AP and lateral radiographs of 18-month-old girl with Paley type 3b1 FH. She has a rocker bottom foot and obvious subtalar coalition malunion. (**b**) Intraoperative fluoroscopic views showing splitting of subtalar coalition with an osteotome (**left**) and after shortening of tibia relative to fibula with plate fixation (SHORDT) combined with subtalar coalition malunion reduction and pinning. (**c**) Radiograph showing lengthening of tibia with computer dependent external fixator at the same surgery as the SUPERankle procedure. A hemiepiphysiodesis plate was also placed to treat the distal femoral valgus. (**d**) Final radiographs after removal of external fixator and after correction of proximal tibial valgus with a hemiepiphysiodesis plate. Note the stable appearance of the ankle joint and the plantigrade foot position. (**e**) Radiographic sequence of extramedullary lengthening with medially placed Precice nail (Nuvasive Specialized Orthopedics, California) (**left**). There is a Simple Locking IntraMedullary (SLIM) rod (Pega Medical, Montreal, Canada) and the fibula is fixed with tibio-fibular screws. The foot is fixed with a temporary extra-articular spanning screw from the foot to the tibia anterior to the ankle joint. A 5 cm lengthening was performed causing axial deviation into valgus bending the SLIM rod (**middle**). To correct the valgus, a plate was inserted laterally, and the extramedullary nail removed, after first decompressing the peroneal nerve, performing an anterior compartment fasciotomy and cutting the fibula proximally (**right**).

The SUPERankle procedure (Systematic Utilitarian Procedure for Extremity Reconstruction) was first developed by the senior author (D.P.) in 1996 to treat fixed equino-valgus or equino-varus deformities [21] (Figure 5, Figure 6 and Figure 7). This is common in Paley type 3 FH, where there is fixed equino-valgus and in Paley type 4 FH, where fixed equino-varus is present. The SUPERankle involves fibular anlage resection with a supramalleolar shortening and realignment osteotomy of the tibial and/or a subtalar osteotomy in order to achieve a plantigrade and stable foot and ankle. The surgical technique and specific SUPERankle variations designed for Paley FH classification type 3, its subtypes and type 4 are described in detail in his publication in 2016 [18].

Since its original description, the SUPERankle procedure has evolved, with the senior author (D.P.) modifying it in 2008 to perform a shortening osteotomy of the distal tibia osteotomy instead, to avoid lengthening tendons. This modification avoided loss of push-off strength and development of a supination midfoot deformity that can occur with weakness that results from lengthening of the Achilles and peroneal tendons, respectively [17].

The surgical approach has been modified over time as well. Originally the SUPERankle procedure was performed through a lateral approach to achieve both the fibular anlage resection as well as the tibial deformity correction. However, a medial approach is now preferred. Only the distal fibular anlage requires routine resection, and the proximal fibular anlage is not routinely released. Although counterintuitive, as from a medial approach it would seem the tibia would block exposure of the fibular anlage for resection, the interval posterior to the tibia and anterior to the posteromedial neurovascular bundle is easy to expose, leading directly to the fibular anlage. Once excised, the remainder of the procedure involving the tibial osteotomy is much easier to complete through a medial approach than the original lateral approach.

## 6. Knee Valgus Deformities

Independent of the tibial diaphyseal valgus, the knee joint in FH often presents with valgus orientation. This genu valgum can originate in the proximal tibia, distal femur or both. Given the foot and ankle valgus instability (dynamic or fixed) that is common in FH, the ankle cannot compensate for a valgus knee. Not only is genu valgum problematic in terms of overall limb alignment, but if untreated can cause recurrence of the ankle deformity and hence it is imperative to detect and treat when present. Determining whether the genu valgum originates from the proximal tibia or distal femur is determined by measuring the lateral distal femoral angle (LDFA) and medial proximal tibia angle (MPTA) [22]. Guided growth via a hemi-epiphysiodesis device placed on the medial distal femur in cases of decreased LDFA and/or the medial proximal tibia in cases of increased MPTA will adequately correct the genu valgum. However, recurrent knee valgus in the proximal tibia is common in FH and likely related to the Cozen phenomenon [23] and repeat hemi-epiphysiodesis may be necessary. If tibial lengthening is performed and the knee valgus originates from the proximal tibia, then the deformity can be corrected through the lengthening osteotomy of the proximal tibia instead of hemi-epiphysiodesis. In this scenario, recurrent knee valgus in the tibia can be prevented by intentionally deforming the tibia into 10–15° of varus at the end of the lengthening to compensate for the expected rebound valgus.

## 7. Knee Ligaments

**Figure 8:**
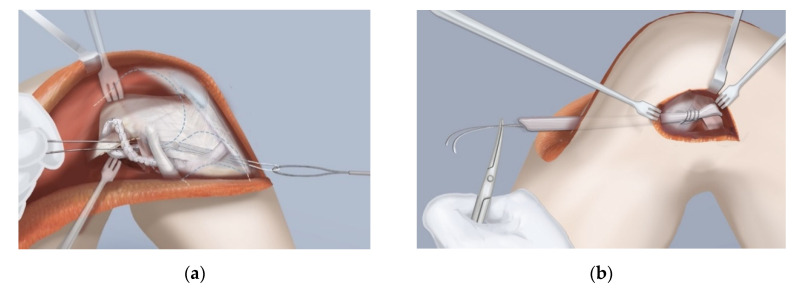
Illustrations of the SUPERknee procedure which consists of two extra-articular ligaments and one intra-articular ligament to replace the anterior cruciate ligament (ACL) and posterior cruciate ligament (PCL). Using the iliotibial band an extra-articular ACL ligament running from Gerdie’s tubercle, passing under the lateral collateral ligament, and around the intermuscular septum (MacIntosh) and an intra-articular ACL passing over the top of the femoral condyle and through the intercondylar space that was widened by a notchplasty and exiting through a tibial tunnel are created (**a**). An extra-articular PCL (reverse MacIntosh) is created from another limb of the iliotibial band running under the patellar tendon, around the medial retinaculum and around the adductor magnus tendon to be sutured back to itself (**b**).

Cruciate ligament hypoplasia or deficiency of the knee is common in FH. Most patients will have some degree of instability in the anterior or posterior direction. Pate et al. concluded that knee related activities appear to be worse in children with FH [24]. The SUPERknee procedure was designed by the senior author as a comprehensive procedure to address the congenital deficiency and deformities in the knee often present in both FH and congenital femoral deficiency (CFD) [25,26,27] (Figure 8). If the knee instability in FH is symptomatic or if the knee remains subluxated with the knee in full extension, then ligament reconstruction with the SUPERknee using either the iliotibial band or allograft tendon may be necessary. As opposed to femoral lengthening in congenital femoral deficiency, when lengthening the tibia in FH stabilization with the SUPERknee procedure is not mandatory before proceeding with lengthening if the knee does not subluxate in full extension and is otherwise asymptomatic. Due to dysplasia of the distal femur in CFD and FH a notchplasty is needed to protect the intra-articular anterior cruciate ligament ACL reconstruction from getting pinched by the femoral condyles. This is a more recent addition to the procedure [28].

## 8. Toe/Metatarsal Surgery

Syndactyly is common in FH. Reconstructing the syndactyly if between the first and second metatarsal with release and skin grafting can be beneficial, especially for shoe and sandal wear. Syndactyly of lesser toes rarely needs surgical intervention. Deficiency or complete absence of one or more toes is common as well. Historically, the best prognostic factor in FH was the foot deformity itself, with many surgeons recommending amputation if there was an absence of two or more metatarsals [14]. However, the senior author’s results do not support this approach [29,30,31]. As long as the foot is plantigrade, the foot is usually highly functional regardless of how many rays are present.

## 9. Lengthening

Prior to lengthening the tibia, any ankle instability and deformity should be addressed. Paley type 1 FH by definition have stable ankles and typically do not require any foot or ankle surgery before lengthening. Most Paley type 2 FH benefit from the SHORDT procedure to correct ankle valgus and procurvatum, stabilizing the ankle. The SHORDT procedure can be performed prior to or combined with the tibial lengthening. If the ankle deformity and LLD in Paley type 2 FH are not severe and no lengthening is planned until later childhood or adolescence, then the SHORDT procedure can be delayed until the patient is older.

In contrast, Paley type 3 and 4 FH have fixed foot and ankle deformities and should be corrected early to allow ambulation with a plantigrade foot and to promote proper shoe wear. The SUPERankle procedure must be performed prior to any lengthening or can be combined with a tibia lengthening if performed with an external fixator that incorporates the foot to protect the foot and ankle during lengthening. Ideally, the SUPERankle procedure is performed in children between 18 and 24 months of age, especially when combined with the 1st lengthening. The tibial osteotomy for lengthening is performed in the proximal tibia, distal to the proximal pins. Soft tissues can be difficult to close in some cases of severe deformity in which a significant amount of bone shortening is required as part of the SUPERankle procedure. In these cases, application of the lengthening frame and proximal tibial osteotomy is staged 2–3 weeks later once the soft tissues have healed.

If tibial lengthening is to be combined with the SHORDT procedure in Paley type 2 FH, then typically no foot fixation is required, and ankle motion can be protected with cast initially for 6 weeks until the distal tibial osteotomy is healed. Then, a nighttime stretching AFO brace is used with physical therapy until the lengthening is complete.

The lengthening goal at the time of surgery is tied to several factors, most importantly anticipated discrepancy at skeletal maturity. Developing a reconstructive life plan is important in FH treatment, in order to address all deformities and develop a plan for limb equalization. A reconstructive life plan is a highly individualized plan that corrects all deformities and chooses timing and goal of each limb equalization surgery to achieve limb equalization in as few surgeries as possible.

Under the age of 4 years old, lengthening up to 5.0 cm can safely be performed without concern for growth inhibition [29]. In the tibia, a 7-day latency is commonly used, followed by 0.75 mm of total lengthening per day, divided into 3–4 sessions. This is the author’s preferred rate of lengthening to ensure fewer complications of failure of bone formation and contractures of the knee, ankle and toe joints. Subsequent tibial lengthening in older children can be performed up to 8.0 cm of lengthening. This would allow one 5.0 cm lengthening at age 4, one 8.0 cm lengthening at age 8 and another 8.0 cm lengthening at age 12, achieving 21.0 cm (5 + 8 + 8 cm). If additional equalization is required, epiphysiodesis of the opposite tibia (timed with the Paley multiplier formulae) can be performed at the appropriate age-specific time for up to 5.0 cm. This allows leg length equalizations up to 26.0 cm to be achieved with three lengthenings (21 cm) plus an epiphysiodesis (5 cm).

**Figure 9:**
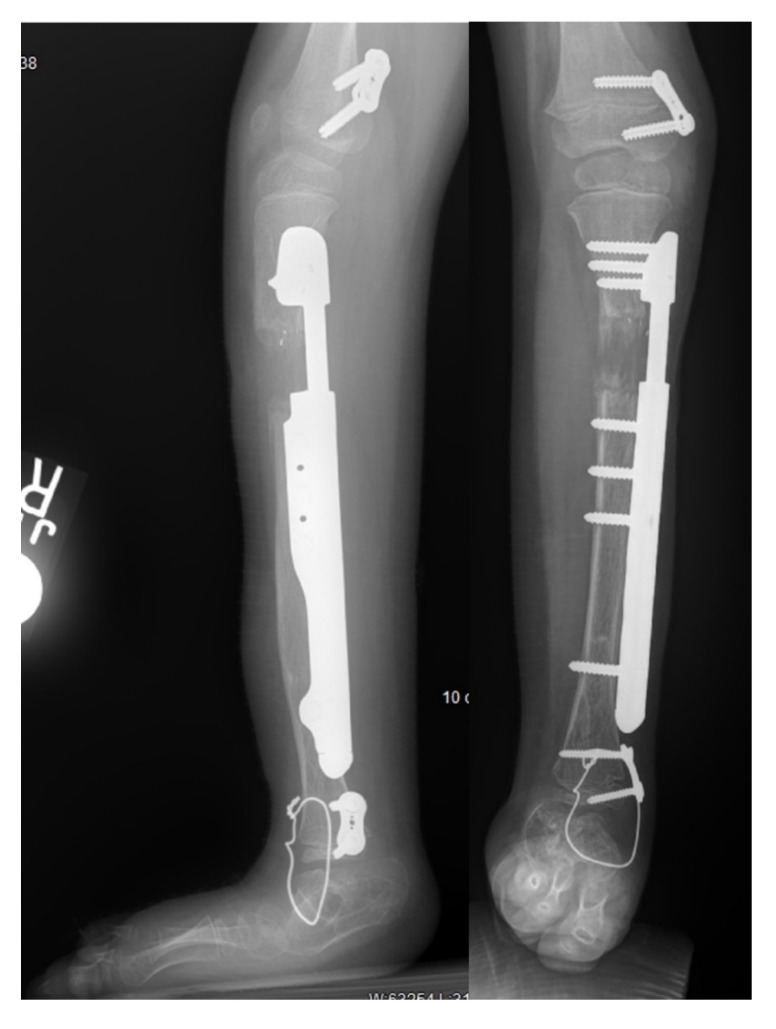
AP and lateral radiographs of a 4-year-old girl who had a previous SUPERankle procedure and one 5 cm lengthening with an external fixator. She had a Precice plate applied to the medial side of the tibia. To prevent equinus the foot was tethered anteriorly with a temporary arthrodesis wire. The Precice plate (Nuvasive Specialized Orthopedics, Aliso Viejo, CA, USA) can lengthen up to 4.5 cm.

With the recent advancement of internal lengthening devices, there is a trend to perform lengthenings internally if possible. External fixators still play an important role in tibia lengthening, especially in young children where physeal and bone diameter or length prevent insertion of a rigid nail. All-internal lengthening, with an extramedullary implant position is an emerging option in patients who would traditionally require an external fixator for lengthening in the femur and tibia. Extramedullary implantable limb lengthening (EMILL) is a new technique, first performed by the senior author in 2015 [32], that uses an implantable nail attached to the bone like an internal-external fixator. EMILL has been shown to be a safe technique to lengthen the femur or tibia in patients in which an external fixator would otherwise be necessary [32,33,34]. There were 4 patients who underwent EMILL for tibial lengthening reported by Shannon et al. [34]. None had axial deviation. One had a locking screw breakage and one had a screw head erode through the thin skin on the medial side of the leg. Although there were no reported axial deviations, the authors have subsequently had cases of axial deviation with tibial EMILL (Figure 7). The senior author, in collaboration with Nuvasive Specialized Orthopedics has continued the evolution of the Precice mechanism to develop an implantable limb lengthening plate called the Precice plate. The Precice plate device was specifically designed for EMILL and has now was cleared by the FDA in December of 2019 (Figure 9). It has been used by the authors in 20 cases and will be the subject of a future report. A lengthening plate further expands the indications for EMILL and the ability to utilize implantable lengthening for younger children [35].

Lengthening of the tibia with an all-internal technique does carry additional risks of developing an equinus contracture compared to lengthening with an external fixator in FH. External fixators can be applied across the ankle to incorporate the foot, thus allowing control of the foot and ankle during lengthening to prevent equinus. All patients undergoing internal tibial lengthening are required to use ankle-foot orthosis when sitting or lying around and undergo daily physical therapy to prevent equinus. The lengthening goal in FH should be restricted to 5.0 cm or under and lengthened at a rate of 0.75 mm/day to avoid increased risk. In higher-risk tibial lengthenings, the senior author (D.P) developed a technique in 2003, inserting a temporary extraarticular ankle stabilization (EAAS) screw between the calcaneus and tibia to prevent equinus [36]. Inserting an extraarticular calcaneo-tibial screw is a safe and powerful technique that can be used in skeletally mature patients with FH to adequately maintain ankle dorsiflexion during the lengthening phase.

Another strategy to mitigate complications in all-internal tibial lengthening of FH is to perform more frequent and shorter lengthening. All-internal lengthenings can more easily and frequently be repeated than lengthening with external fixation, reducing the need to achieve large single-stage lengthening. Soft tissue trauma from implantable lengthening is less than external fixation lengthenings, resulting in a shorter recovery period. This can allow the time between lengthening to be reduced to 2 to 3 years. In some instances, the internal lengthening device can remain inside the patient between lengthenings and can be reactivated with a small percutaneous osteotomy to perform another lengthening. For example, if an internal lengthening nail with 8 cm of lengthening potential is inserted for a goal of 8 cm of lengthening, it would be safer to divide the lengthening into two 4 cm lengthenings 2 years apart, instead of attempting 8 cm in a single stage. This would allow one to utilize only 4 cm of the implant’s potential at the first lengthening and then to return 2–3 years later and with a small percutaneous osteotomy reactivate the nail and gain an additional 4 cm. Although this does increase the number of surgeries, advantages include less soft tissue trauma, better cosmesis, shorter recovery period and decrease risk of large lengthening complications such as joint instability, contractures and neuropathy. More frequent and shorter lengthenings is an increasingly used strategy in all-internal lengthening to mitigate lengthening complications.

## 10. Results

Historically, the combination of a leg length discrepancy with the foot and ankle deformities in FH led to controversy in recommending reconstruction versus amputation. Today, most agree that cases of mild to moderate leg length discrepancy combined with a mild to moderate foot deformity in FH benefit from reconstruction with lengthening. However, in cases with more significant leg length discrepancies and foot and ankle deformities (as commonly found in Paley type 3 and 4 FH), some surgeons recommend amputation over reconstruction because of historical failure to obtain acceptable outcomes after limb lengthening [37,38,39,40].

A close examination of poor results in several series show that the main factor associated with unacceptable outcome in reconstruction is recurrent or residual foot deformity [31,38,40,41]. Naudie et al. [37] retrospectively reviewed 22 patients with fibular hemimelia who underwent amputation or limb lengthening to compare outcomes. Twelve patients had amputation with prosthetic fitting and ten had lengthenings using the Ilizarov technique. They concluded that amputation was preferred over lengthening due to poor outcomes in the lengthening group, largely due to residual or recurrent foot and ankle deformities. Similarly, Choi et al. [38] retrospectively reviewed outcomes between amputation and lengthening in 43 patients with fibular hemimelia. Thirty-two patients had Syme or Boyd amputation in contrast to 11 patients that underwent lengthening with the Wagner technique. They found that satisfactory results were achieved in all but one of the patients with mild FH and all of the higher-grade cases of FH had poor results, which they concluded was due to the rigid, uncorrected equino-valgus foot deformity. This led them to conclude amputation was preferred in more severe cases of FH.

In contrast, most series that have reported satisfactory outcomes, the foot deformity was corrected with a resultant stable plantigrade foot, even if ankle arthrodesis is ultimately required [42,43,44,45,46,47]. Catagni and Guerreschi [42] reported 89 patients who underwent reconstruction and lengthening and were classified according to the Dal Monte classification [13]. There were 32 patients with grade 1 FH who underwent lengthening, all of which achieved equal leg lengths and a plantigrade foot. There were 37 patients with grade 2 FH, in which 35 achieved a plantigrade functional foot. The grade 3 FH group consisted of 20 patients who all underwent reconstruction and lengthening resulting in 16 plantigrade and stable feet. All patients were satisfied with their functional outcome and most of the patients could pursue athletic pursuits such as biking and swimming. However, this was more limited in the grade 3 patients. Gait analysis data from Johnson and Haideri [48] further support the importance of achieving a plantigrade foot. In their gait analysis they found that when a plantigrade and well aligned tibia is achieved in fibular hemimelia reconstruction, knee flexion strength and push-off ankle strength are better compared to those who undergo Syme’s amputation.

A recent meta-analysis by Elmherig et al. [49] in 2020 of seven retrospective studies also highlights the importance of correcting the foot deformity. The study included 211 patients with fibular hemimelia in which 120 underwent amputation and the other 91 patients underwent limb reconstruction and lengthening. The authors found that patients had less complications and were more satisfied with amputation in older studies, largely due to foot pain and recurrent unresolved ankle deformities in the limb reconstruction group. They found that more recent studies have better outcomes with reconstruction, due to the advances in limb reconstruction techniques designed to correct the foot deformity, such as the SUPERankle procedure. It is clear from the literature that even if limb equalization is achieved, if the foot is not successfully corrected or the foot deformity recurs, the final outcome will not likely be satisfactory.

Reconstructive techniques for fibular hemimelia have advanced significantly since many of these studies have been published, especially in treating the foot and ankle deformity. The SUPERankle and SHORDT procedures were designed specifically to correct the underlying deformity and stabilize the foot and ankle. With these newer reconstructive techniques, the senior author has been able to achieve excellent functional results with reconstruction and lengthening in most of his cases. His series in 2011 [31] included 38 patients with FH, in which 94.7% (36/38) of his patients achieved excellent functional results with limb equalization achieved. All adults from this series were employed and most of these patients were involved in recreational or competitive activities. Although complications arise, most do not lead to major sequelae and those that do can usually be resolved surgically [50].

Despite historic literature showing mixed results, recent literature has shown improved results in limb reconstruction for fibular hemimelia using modern techniques such as the SUPERankle procedure, supporting that most severe cases can be treated successfully with modern techniques. Birch et al. compared 20 children with severe FH treated by primary amputation at one institution with 22 children treated at another institution by reconstruction with the SUPERankle procedure followed by limb lengthening [51]. All patients and parents in the study completed surveys including quality of life (QoL) in addition to physical testing that included instrumented gait analysis and timed 50-yard dash. Family of the children in the amputation group were more ethnically diverse and had lower socioeconomic and education levels than families of the limb reconstruction group. Psychologically, they found no significant difference between the groups and functionally both groups showed similar performance in gait analysis and timed 50-yard dash without statistical difference. All patients and parents were satisfied with their outcome, irrespective of choosing amputation or limb reconstruction and would choose the same treatment method again. Ultimately, they concluded that limb reconstruction is a viable option and one “must weigh life-long prosthetic requirements against significantly greater number of surgical interventions for limb salvage and reconstruction.”

Kulkarni et al. [52] in 2019 studied 29 patients with FH, of which 27 were treated with reconstruction and followed for an average duration of 9.37 years. Cases were classified according to the Paley classification with Paley type 1 FH receiving tibial corrective surgery with lengthening, Paley type 2 underwent distal tibial realignment with guided growth versus osteotomy, Paley type 3 underwent SUPERankle procedure with subsequent lengthening and Paley type 4 underwent the specific SUPERankle variation. In their study, the majority (77%) had excellent or good outcomes at final follow-up according to the Association for the Study and Application of Methods of Illizarov (ASAMI) scoring system. Additionally, they emphasized treating the foot deformity early, as feet reconstructed before the age of 5 were the less likely to recur. They concluded that “limb reconstruction according to Paley classification, is an excellent option in the management of fibular hemimelia.”

## 11. Conclusions

The outcome in reconstructive surgery for FH is dependent on obtaining a plantigrade and stable foot. The SHORDT and SUPERankle procedures are type-specific procedures to correct the foot deformity in moderate to severe cases of FH. Leg length discrepancy is successfully treated with serial lengthening with possible epiphysiodesis. New technological advances in implantable lengthening devices and surgical techniques (EMILL) are allowing more lengthenings to be performed all-internal, making them easier and more tolerable for patients. In light of these results, all patients should be given the option of surgical reconstruction versus amputation.
